# Recent Progress of Tactile and Force Sensors for Human–Machine Interaction

**DOI:** 10.3390/s23041868

**Published:** 2023-02-07

**Authors:** Jiandong Xu, Jiong Pan, Tianrui Cui, Sheng Zhang, Yi Yang, Tian-Ling Ren

**Affiliations:** 1 School of Integrated Circuits and Beijing National Research Center for Information Science and Technology (BNRist), Tsinghua University, Beijing 100084, China; 2 Shenzhen International Graduate School, Tsinghua University, Shenzhen 518055, China; 3 Center for Flexible Electronics Technology, Tsinghua University, Beijing 100084, China

**Keywords:** tactile sensor, force sensor, HMI, VR/AR, feedback system

## Abstract

Human–Machine Interface (HMI) plays a key role in the interaction between people and machines, which allows people to easily and intuitively control the machine and immersively experience the virtual world of the meta-universe by virtual reality/augmented reality (VR/AR) technology. Currently, wearable skin-integrated tactile and force sensors are widely used in immersive human–machine interactions due to their ultra-thin, ultra-soft, conformal characteristics. In this paper, the recent progress of tactile and force sensors used in HMI are reviewed, including piezoresistive, capacitive, piezoelectric, triboelectric, and other sensors. Then, this paper discusses how to improve the performance of tactile and force sensors for HMI. Next, this paper summarizes the HMI for dexterous robotic manipulation and VR/AR applications. Finally, this paper summarizes and proposes the future development trend of HMI.

## 1. Introduction

Tactile and force sensing is important for humans to understand and interact with the external world. Human skin, especially the skin of the hand, can sensitively sense pressure, strain, and bending stimuli. In order to imitate the tactile and force sensing capability of human skin and to record tactile information for practical use, flexible tactile and force sensors were developed in the form of electronic skin [1], electronic fabric [2], smart contact lenses [3], etc. Compared to conventional bulky and rigid devices, flexible tactile and force sensors can be attached to curved and soft surfaces; thus, they are suitable to be used for wearable electronics with high comfort and fitness [4]. Moreover, with the development of material and structural design and micro-nano processing technology, flexible tactile and force sensors have higher sensitivity and lower response time than conventional devices, and some even surpass the performance of the human skin [5]. Flexible tactile and force sensors have been applied to a variety of applications, including health monitoring [6], object recognition [7], intelligent robots [8], human–machine interaction (HMI) [9], etc., where HMI is receiving increasing attention since it serves as a bridge to connect human and robots, devices, or virtual avatars.

During the human–machine interaction process between the user and the machine, the user first enters a signal through the tactile and force sensors; then, the input signal is converted into a directive and transmitted to the machine system, and finally, the machine system carries out a task corresponding to the directive [4]. The tactile and force sensors are the hardware fundamental of an HMI system since they determine the sensitivity, accuracy, and response time of the system to receive input from the user. Commonly used types of tactile and force sensors include resistive sensors, capacitive sensors, piezoelectric sensors, and triboelectric sensors, where resistive sensors have high sensitivity and simple readout, but the power consumption is relatively high; capacitive sensors have low power consumption but are sensitive to electromagnetic interferences; piezoelectric and triboelectric sensors have self-powered sensing properties; and triboelectric sensors can detect not only dynamic but also static tactile signals. Recently, strategies to improve the performance of tactile and force sensors have been proposed, including the enhancement of the linear detection range, sensitivity, wearing fitness, and the capability of multi-dimensional tactile sensing, which have the potential to be applied to HMI applications [5,10,11,12].

With the aid of nanofabrication, tactile mechanisms, and the advanced recognition method, novel HMIs in the form of a keyboard, gear, or touchscreen [13,14,15] and HMI systems for wireless communications [16] have been developed for advanced performance and intelligent interaction. Apart from those conventional HMI forms, novel forms of HMI as electronic skin or smart clothing have been demonstrated with the increasing requirement for wearable HMI applications, especially for robot control. Robot control HMIs consist of multiple tactile and force sensing units to achieve the multi-channel monitoring of human body motion, and the group of signals received by the sensing units is processed and recognized to generate directives to control robots [17]. Especially with the development of the meta-universe, as a window for people to experience the virtual world, the HMI must be combined with the corresponding feedback system to let people immerse in the real experience of the virtual world, such as sports, games, and other fields. Traditional VR/AR technology mostly depends on the sensory perception of glasses and ears. However, as the largest sensory organ of the human body, the skin can feel more feedback such as temperature, vibration, shape, etc. Therefore, more and more researchers develop skin-integrated electronic sensors as the feedback system of immersive VR/AR experiences [9,18,19]. In addition, other wearable electronic device systems such as smart gloves and rings have also been developed for VR/AR technology applications [20]. Recently, novel strategies have been rapidly developed for advanced tactile and force sensors applicable to HMIs, and HMIs with novel structures, system designs, and scenarios are demonstrated for robot control and VR/AR technology. Therefore, it is necessary to provide an overview and give the future outlook of tactile and force sensors for HMI.

This paper reviews the recent progress of tactile and force sensors for human–machine interaction. Section 2 introduces widely used mechanisms of tactile and force sensors for HMIs. Section 3 focuses on the methods to improve the performance of tactile and force sensors for advanced HMIs. Section 4 illustrates the recent achievements of HMIs for robot control applications. Section 5 describes the HMIs for VR/AR applications, and finally, this work is concluded in Section 6.

## 2. Tactile and Force Sensors for HMI

### 2.1. Resistive Tactile and Force Sensors

Resistive tactile and force sensors are widely used in human–machine interfaces for their high sensitivity and simple readout circuits, and they can be applied to sense different forms of forces, including pressure and strain. Resistive tactile and force sensing is based on the change of the contact resistance R of the active layer that is given by:

(1) $$R=\rho \frac{L}{A}$$

where ρ is the resistivity of the active material, L is the length, and A is the contact area that is continuously changed by the force loaded on the sensor. As a result, each resistance value corresponds to a force value.

**Figure 1:**
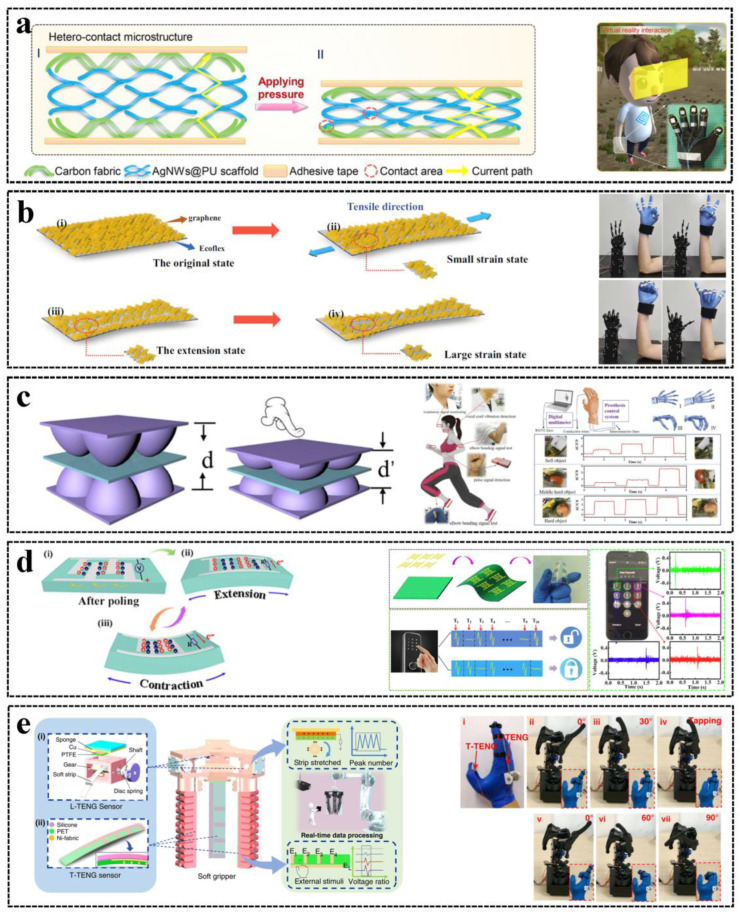
Tactile and force sensors for HMI: (**a**) A resistive pressure sensor for virtual reality. Reproduced with permission [28], Copyright 2019, Elsevier Ltd. (**b**) A resistive strain sensor for robot control. Reproduced with permission [37], Copyright 2022, Springer Nature. (**c**) A capacitive pressure sensor for physiological signal and robot grabbing monitoring. Reproduced with permission [50], Copyright 2020, Elsevier Ltd. (**d**) A piezoelectric pressure sensor for self–powered HMI. Reproduced with permission [60], Copyright 2022, Elsevier Ltd. (**e**) Triboelectric sensors for robot control. (i) and (ii) are the structures of the L–TENG and T–TENG sensors respectively. Reproduced with permission [66], Copyright 2020, Springer Nature.

A resistive pressure sensor has an active layer that is conducive for measurement and elastic for response to pressure, which can be made by coating the conductive nanomaterial, such as nanowires (NWs) [21], reduced graphene oxide (rGO) [22], carbon nanotubes (CNTs), graphene, or MXene [23], on the elastic polymer or by combining the conductive material and the polymer to form the composite film [24]. When pressure is applied, the shape, contact area, and resistivity of the active layer are altered, and the resistance is changed accordingly [25]. Resistive pressure sensors are used to sense and record the contact between the human body (e.g., finger or foot) and the contact surface for HMI applications [26,27]. An AgNW-based resistive pressure sensor was demonstrated by Liao et al. [28], and high sensitivity (>2.6 kPa^−1^), fast response (55 ms drop time and 64 ms rise time), and high stability were achieved by the hetero-contact microstructure for real-time virtual reality applications, as shown in Figure 1a.

Resistive strain sensors as flexible electronic skins consist of nanomaterials on elastic films or directly on human skin, composite polymers [29], or textiles [30]. Graphene [31], CNTs [32], and MXene [33] are widely used nanomaterials of resistive strain sensors. The resistance changes of strain sensors are mainly caused by the piezoresistive effect, geometrical change, connect area change, crack propagation, and tunneling effect [34]. Resistive strain sensors are commonly used for monitoring skin stretching caused by arthrosis or muscle motion by attaching to human skin [17,35], or they are used for intraocular HMIs by attaching to smart contact lenses [36]. Zhou et al. developed a resistive strain sensor based on graphene/Ecoflex composite films [37]. High sensitivity (the gauge factor is 1078.1) and stretchability (650%) were achieved due to the hierarchical wrinkle structure; thus, the sensors can be attached to places that have high motion ranges, such as fingers, for wearable HMI applications, as shown in Figure 1b.

Resistive tactile and force sensors can be designed and fabricated in various forms to adapt to specific scenarios with their simple fabrication, small size, and flexibility. For example, Yang et al. developed a non-printed integrated-circuit textile for body health monitoring [38]. A resistive motion sensor and circuit modules were integrated into a textile, which looks like a normal cloth and can be worn by users. Apart from electronic textiles, many electronic skins use resistive sensors for tactile sensing. Qiao et al. proposed a tattoo-like epidermal resistive sensor for strain sensing [31]. Due to the transferable property of the electronic skin, it can not only serve as a health monitor but can also be attached to objects as artwork. Furthermore, many smart contact lenses (SCLs) utilize resistive sensors to monitor intraocular pressure (IOP) for continuous glaucoma diagnosis. Kim et al. developed an SCL integrated with a circular antenna and a strain sensor for continuous IOP monitoring [39], and the intraocular sensing and output circuits did not have a chip to ensure thin thickness and comfortable wearing.

### 2.2. Capacitive Tactile and Force Sensors

Capacitive sensors are commonly used for tactile sensing because of their high sensitivity and low power consumption, and they can be designed to reduce interferences by temperature fluctuation [40,41]. However, capacitive sensors are sensitive to outer electromagnetic interferences. Most flexible capacitive tactile and force sensors are parallel-plate capacitors, which consist of an elastic medium sandwiched between two electrodes [42], and the capacitance C is given by:

(2) $$C=\frac{\varepsilon A}{d}$$

where A is the contact area, d is the distance between the two electrodes, and ε is the permittivity of the medium. The distance d is reduced with the increase of the applied pressure; thus, the capacitance is continuously increased with the increment of the pressure [43]. Another type of capacitive pressure sensor is called an interdigital pressure capacitor, which has also been reported for tactile sensing [44]. Various elastic materials, including Polydimethylsiloxane (PDMS) [45], Ecoflex [46], and Polyurethane (PU) [47], are utilized as the elastic medium of capacitive sensors. The elastic medium of capacitive sensors has many types of structures, mainly to enhance sensor sensitivity. A simple structure is a spacer between two electrodes to make an air gap at the medium of a capacitive sensor. Joo et al. introduced PDMS spacers between two electrodes and developed a highly sensitive (9.9 kPa^−1^ in 0–0.6 kPa and 0.6 kPa^−1^ in 0.6–6.6 kPa) capacitive sensor [48]. Since most polymers have higher permittivity (high k) than air, polymers doped with high k or even conductive fillers can further enhance the capacitive sensor performance, according to Equation (2). Ha et al. developed a capacitive pressure sensor with a CNT-doped Ecoflex porous nanocomposite [49], and a high sensitivity and large detection range (3.13 kPa^−1^ in 0–1 kPa and 0.43 kPa^−1^ in 30–50 kPa) were achieved due to the high k composite. Moreover, the surface microstructure is a popular strategy of capacitive sensor medium fabrication because of its simple fabrication process, effective sensitivity increment, and variety of microstructure shapes with different sensitivities, detection ranges, and response times that designers can test to adapt to different scenarios. An electrode surface patterned capacitive pressure sensor was proposed by Xiong et al. [50], and an ultra-high sensitivity of 30.2 kPa^−1^ was demonstrated due to the convex microarray surface structure. Physiological signal and robot hand grabbing motion monitoring was carried out to validate the performance of the capacitive sensor and the potential of the sensor to be applied to HMIs, as shown in Figure 1c.

Both resistive and capacitive sensors can sense static and dynamic tactile signals, enabling them to recover the applied force signals in real HMI applications. Apart from the touch sensing function that is similar to resistive sensors, capacitive sensors respond to the non-contact signal when the body approximates the sensor, which can be classified as a form of generalized tactile signal that is useful for HMI applications. The human can be seen as a conductor connecting to the ground, and when part of the human body, such as a finger, approximates the anode of a capacitive sensor, an equivalent capacitance is formed between the human body and the anode that is in parallel with the anode and cathode of the capacitive sensor, resulting in the increase of the total capacitance [51]. Based on that mechanism, capacitive sensors for contact and non-contact tactile sensing were proposed by Li et al. [52], and a smart pad for proximity recognition was demonstrated.

### 2.3. Piezoelectric Tactile and Force Sensors

Piezoelectric tactile and force sensors utilize the piezoelectric effect that an electric field is generated by the dipole separation in the piezoelectric material caused by the pressure applied on the surface [53]. The piezoelectric effect is described by the constitutive piezoelectric equations given by [54]:

(3) $$\left[\begin{array}{c} S\\ D \end{array}\right]=\left[\begin{array}{cc} s^{E} & d^{t}\\ d & \varepsilon^{T} \end{array}\right]\left[\begin{array}{c} T\\ E \end{array}\right]$$

where S, T, E, and D are the strain, stress, electric field, and electric displacement matrices, respectively, $s^{E}$ is the compliance tensor at the constant electric field, $\varepsilon^{T}$ is the dielectric constant tensor at constant stress, and d is a piezoelectric constant tensor ($d^{t}$ is the transpose of d). Based on the piezoelectric effect, the mechanical signal of tactile and force is converted to an electric signal that is output without an electric energy source; thus, piezoelectric tactile and force sensors are self-powered.

ZnO [55], PZT [56], and polyvinylidene difluoride (PVDF) [57] are commonly used for piezoelectric tactile and force sensors. For example, Yan et al. proposed a PZT-based cellular sensor array for biomedical monitoring, and the system was self-powered due to the energy harvesting property of the piezoelectric sensor [58]. Instead of using a single piezoelectric material, piezoelectric sensors utilizing composite piezoelectric materials were demonstrated to combine superior piezoelectric and mechanical properties. For example, Tian et al. developed a piezoelectric force sensor based on baklava-structured PZT/PVDF composites, and the piezoelectricity was enhanced due to the potential accumulation and the synergistic effect in the structure [59]. The open-circuit voltage was 2.51 V, and the short-circuit current was 78.43 nA. Recently, novel piezoelectric materials were developed for piezoelectric force sensing. Lv et al. proposed a piezoelectric sensing system using Sm-doped Pb(Mg1/3Nb2/3)O3-PbTiO3 (Sm: PMN-PT) film, and a high open-circuit voltage of 6 V and short-circuit current density of 150 μA/cm^2^ were achieved due to the ultrahigh piezoelectric coefficient (d33 = 380 pm/V) of the Sm: PMN-PT film [60]. Self-powered human–machine interactions based on the Sm: PMN-PT sensor were demonstrated, as shown in Figure 1d. By applying piezoelectric sensors to human–machine interactions, the use of a battery can be saved and the device is more portable [61]. However, since electric energy is generated by the change of the load, the use of piezoelectric sensors is limited in dynamic sensing.

### 2.4. Triboelectric Tactile and Force Sensors

Triboelectric sensors for tactile sensing are based on triboelectric nanogenerators (TENG) that were first reported in 2012 [62] with the principle to convert irregular mechanical energy into available electrical energy. The fundamental working mechanism of TENG is based on the coupling effect of the contact electrification and electrostatic induction happening during the contact and separation processes between two materials with different electronegativities [16]. Firstly, a movable triboelectric layer such us triboelectric material or human skin is left from an electrode; then, the movable layer contacts the electrode and causes a surface charge transfer. Then, the movable layer is left again, and the induced charges at the electrode are decreased to balance the voltage with the ground. The process is repeated, and dynamic signals are generated [63]. The types of material used for triboelectric tactile sensors include Polytetrafluoroethylene (PTFE), PDMS, Polyvinyl chloride (PVC), etc., for electron acceptor materials, and skin, PU, Indium tin oxide (ITO), cotton, etc., for electron donor materials, and materials should be properly chosen to guarantee the correct direction of electron transfer [63,64]. Furthermore, novel materials used for triboelectric tactile sensors were demonstrated for advanced performance, including PAN@ZIF-8 nanofibers that can improve the amount of charges generated during electrification [16].

In addition to being self-powered like piezoelectric sensors, triboelectric sensors can sense static and dynamic tactile signals by changing the circuit configuration [65]. Apart from recently reported triboelectric HMI keyboards [13], handwriting e-skin [15], and 3D control gears [14], flexible triboelectric sensors are widely used in many parts of the human body to collect tactile signals for human–machine interaction, including gesture control [66], eye motion monitoring [67], and foot motion control [68]. For example, Jin et al. proposed a tactile TENG (T-TENG) sensor and a length TENG (L-TENG) sensor for soft robots and robot manipulation by gesture control [66]. Using the T-TENG sensor attached to the thumb that could sense the sliding, contact position, and area, as well as the L-TENG sensor to sense the bending of the finger, a robot control HMI was demonstrated, as shown in Figure 1e. The triboelectric sensor can respond to slight tactile signals such as eye motion; thus, Pu et al. developed a mechanosensation TENG (msTENG) sensor that was mounted on the arms of glasses and could respond to eye motion signal, and a hands-free typing system based on the sensor was demonstrated [67]. Apart from hand and eye motion control, a foot control HMI was also developed based on the triboelectric sensor. Zhang et al. proposed triboelectric intelligent socks for foot-control virtual reality [68]. The thin T-TENG sensor used patterned frustum structures so that a high sensitivity (about 1.2 V/kPa within 42 kPa) and large detection range (>200 kPa) were achieved to realize foot tactile sensing.

### 2.5. Other Types of Tactile and Force Sensors

Apart from typical sensors, other types of tactile and force sensors were reported for HMI. An electrical impedance tactile sensor is based on the measurement of electrical impedance change when force is applied [69,70]. An optical fiber tactile sensor is based on the transmittance alternation of different light wavelengths by the applied force, and an optical microfiber for HMI has been developed accordingly [71]. A magnetic tactile sensor consists of an active layer where the density and distribution of magnetic particles are changed by the applied force, and a Hall sensor captures the magnetic field change [72]. The other type of magnetic tactile sensor consists of a magnetic layer that can be deformed by pressure and a giant magneto-resistive material layer that senses the deformation of the magnetic layer [73]. Moreover, different tactile sensing mechanisms can be combined for multifunctional tactile sensing, e.g., a heterogeneous tactile sensor able to sense stretching, bending, and compression individually was proposed by combining optical, microfluidic, and piezoresistive sensing mechanisms [74]. In addition to touch tactile interaction, proximity interaction is regarded as a generalized form of tactile, and sensing mechanisms, including capacitive [52], nearby charge induction [75], optical fiber [71], and magnetic field sensing [73], are utilized to realize non-contact tactile HMIs. Typical tactile and force sensors for human–machine interaction developed recently are summarized in Table 1.

## 3. Performance Improvement of Tactile and Force Sensor for Advanced HMI

### 3.1. Linear Detection Range

**Figure 2:**
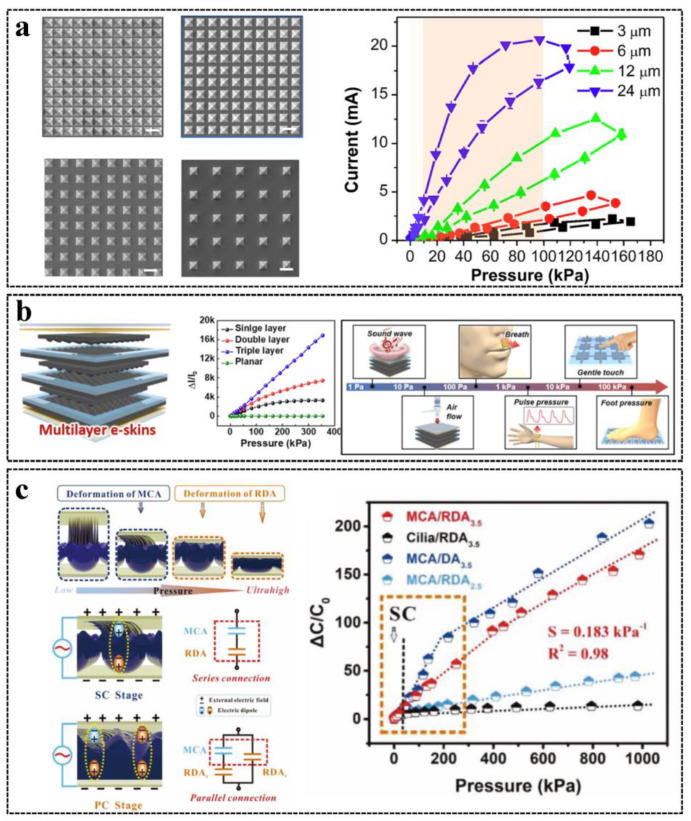
Strategies of tactile and force sensors used to increase the linear detection range: (**a**) Illustration of the trade–off between sensitivity and linear detection range. Reproduced with permission [10], Copyright 2020, American Chemical Society. (**b**) A multilayered ferroelectric sensor that has a larger linear detection range than single and double–layer sensors. Reproduced with permission [86], Copyright 2018, American Chemical Society. (**c**) A hybrid dielectric for capacitive and triboelectric sensors that has an MPa linear detection range. Reproduced with permission [85], Copyright 2021, Wiley–VCH.

The linear detection range is a key specification of tactile and force sensor performance. A tactile sensor that has a large linear detection range can preserve high-pressure resolution over the detection range and can facilitate calibration and data processing, which have the potential for advanced HMI applications [83,84]. Although linearity can be achieved by changing the tactile sensor structure, there is a trade-off between the sensor linearity and sensitivity, since the structure change may increase the linear range but decrease the sensitivity [85]. The structure adjustment to change the linear detection range and sensitivity was illustrated by Ma et al. [10], as shown in Figure 2a. With the increase of the pyramid spacing of the resistive pressure sensor, the sensitivity is increased, but the linear range is decreased. To balance sensitivity and linear range, 12 μm spacing was chosen, and ∼0.34 kPa^−1^ sensitivity and 10–100 kPa linear range were demonstrated. In order to achieve high linearity as well as a large linear detection range, different strategies have been proposed. A flexible ferroelectric sensor was proposed by Lee et al. with a multilayer interlocked micro-dome geometry with high sensitivity (47.7 kPa^−1^) and a large linear range (0.0013–353 kPa) [86], as shown in Figure 2b. Because the pressure is distributed to each layer, the multilayer geometry can increase the linearity, and because the applied stress at small spots is concentrated and amplified due to the interlocked micro-dome structure, the sensitivity is enhanced as well. Furthermore, a hybrid dielectric for capacitive and triboelectric tactile sensors achieving an ultrawide linear detection range (up to 1000 kPa) and high sensitivity (0.314 kPa^−1^) was reported by Ji et al. [85], as shown in Figure 2c. A high linear range was realized because the low-permittivity (low-k) micro-cilia array (MCA) and high-k micro-dome array (RDA) can be converted from series to parallel connections as the pressure is increased, and the main deformation layer is different for different pressure ranges (MCA is deformed largely in low pressure, and RDA is deformed largely in high pressure).

### 3.2. Detection Sensitivity

**Figure 3:**
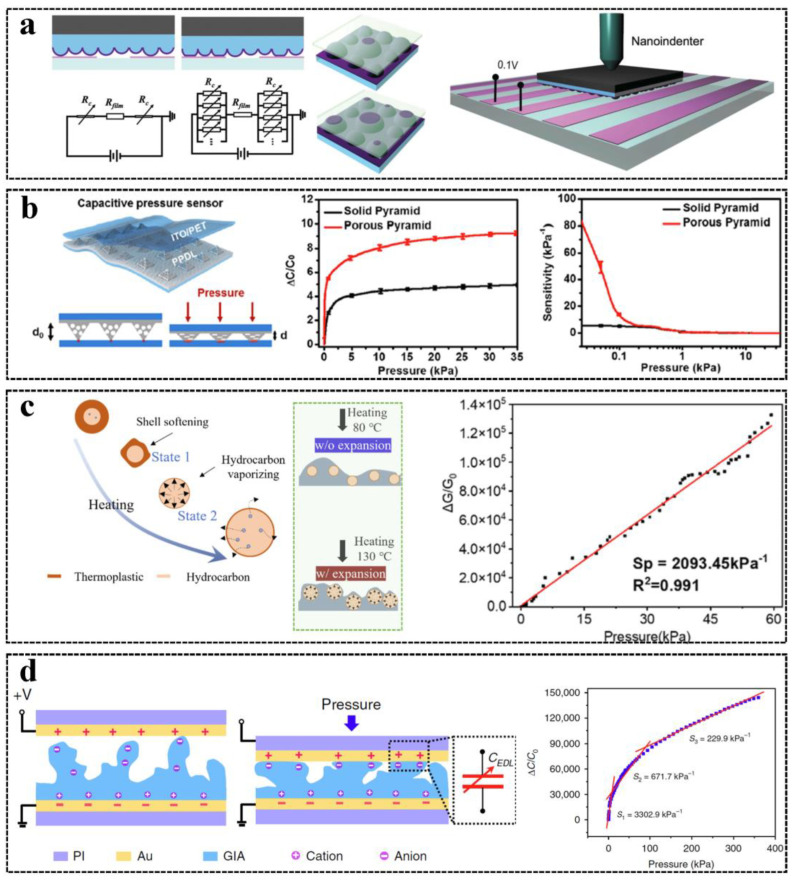
Strategies of tactile and force sensors to increase sensitivity: (**a**) A resistive pressure sensor with irregular surface microstructure enhances the sensitivity. Reproduced with permission [91], Copyright 2016, Wiley–VCHgmb. (**b**) A flexible capacitance sensor using porous and pyramid structures. Reproduced with permission [41], Copyright 2019, American Chemical Society. (**c**) A resistive sensor utilizing thermally expandable microspheres. Reproduced with permission [5], Copyright 2022, Elsevier Ltd. (**d**) An iontronic capacitive sensor with graded–intrafillable architecture. Reproduced with permission [96], Copyright 2020, Springer Nature.

Sensitivity is an important performance parameter of tactile and force sensors for HMI, and highly sensitive tactile sensors can detect slight tactile signals and have high measurement accuracy [25]. The sensitivity of a pressure sensor is defined by the relative change of the output signal such as resistance, capacitance, and voltage per pressure. For strain sensors, the sensitivity is evaluated by gauge factor (GF, $\delta (\Delta R/R_{0})/\delta \varepsilon$), which is the ratio of the relative change to the mechanical strain [34]. The surface microstructure is a commonly used way to increase sensitivity, and active layers with microstructures have higher deformation and larger contact area change under applied forces. Widely used surface microstructures are cylinders [87], domes [88], pyramids [89], etc. Other than regular microstructures, irregular structures such as rough surfaces processed by abrasive paper [27] or salt and sugar [90] were used to mold the material surface, especially for resistive tactile sensors since the change of the applied pressure does not only alter the equivalent resistance but also changes the equivalent number of resistors between the electrodes, which further enhances the sensitivity [91], as shown in Figure 3a. Moreover, porous [92], nanowire [93], and textile [94] layers have also been demonstrated to increase sensitivity, and sensors combining multiple structure features were reported to further enhance the sensitivity [41,95], as shown in Figure 3b. Most structures are made by molds and have the same shape as the molds, but an ultrahigh-sensitive tactile sensor design utilized the thermal expansion property of a kind of microsphere and heated the mixture of PDMS and microsphere after spin-coating on an abrasive paper to obtain a more elastic composite film with a surface microstructure rougher than the abrasive paper [5], as shown in Figure 3c. The sensors achieved high sensitivity (2093 kPa^−1^) and fast response (<4 ms), which are beyond the performance of human skin (<10 kPa^−1^ and 15 ms response time), and had a large detection range (0.43 mN–60 kPa) and excellent linearity.

The increase in sensitivity is not always beneficial because the increment of sensitivity may cause a decrease in the detection range for capacitive tactile sensors. Therefore, there is a trade-off between the sensitivity and detection range for capacitive tactile sensors. A strategy used to increase sensitivity without decreasing detection range called iontronic tactile sensing was developed, which utilizes the change of the electron double layer (EDL) at the interface between the ionic material. When the pressure is loaded, the contact area is changed, which alters the amount of charge induced at the interface and driftly changes the EDL capacitance. An iontronic capacitive pressure sensor with a microstructure molded by the abrasive paper was reported [96], and it achieved high sensitivity (>220 kPa^−1^) and an ultra-broad-range (0.08 Pa–360 kPa), as shown in Figure 3d. Although iontronic capacitive sensors are not sensitive to humidity change, they are very sensitive to temperature, which limits the application scenarios of iontronic sensors.

### 3.3. Multi-Dimensional Sensing

**Figure 4:**
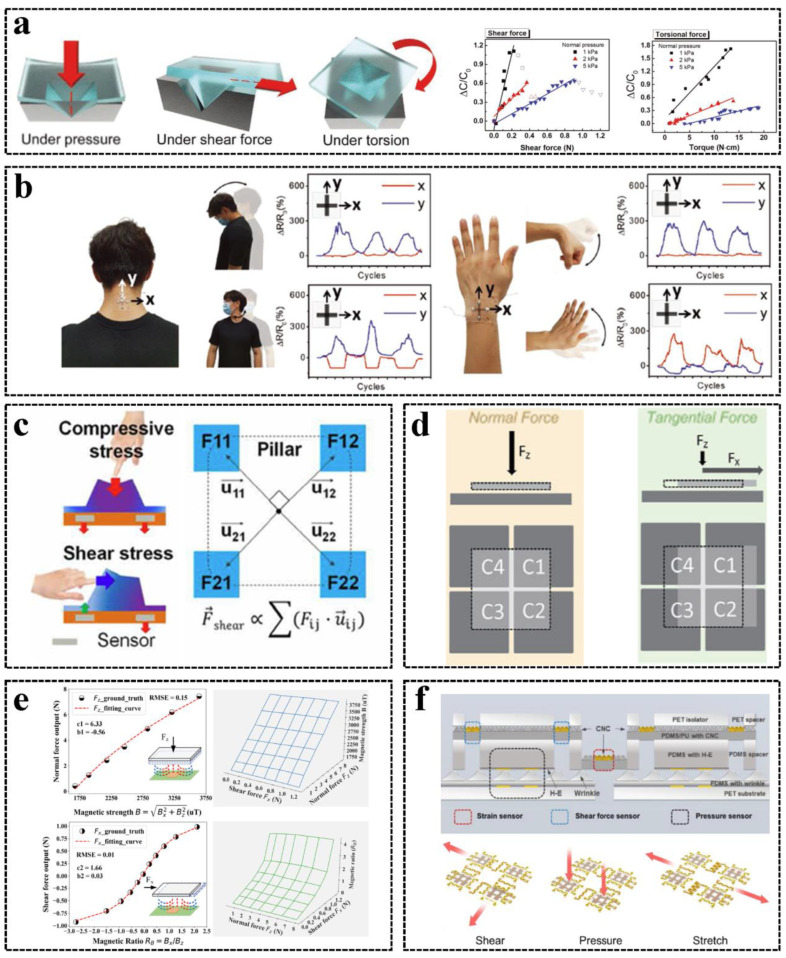
Multi–dimensional tactile sensors: (**a**) Tactile sensors with a single channel. Reproduced with permission [98], Copyright 2018, Wiley–VCH. (**b**) A tactile sensor with two strain sensing units perpendicular to each other. Reproduced with permission [102], Copyright 2019, Wiley–VCH. (**c**) A tactile sensor adds a bump structure for three-dimensional force sensing. Reproduced with permission [112], Copyright 2020, American Association for the Advancement of Science. (**d**) A tactile sensor where four lower electrodes share a common upper electrode. Reproduced with permission [115], Copyright 2014, Wiley–VCH. (**e**) A magnetic multi–dimensional tactile sensor with self-decoupling. Reproduced with permission [72], Copyright 2021, American Association for the Advancement of Science. (**f**) Resistive and capacitive multi–dimensional tactile sensors with the self-decoupling of pressure, shear force, and strain sensing. Reproduced with permission [119], Copyright 2021, Elsevier Ltd.

In addition to single force detection such as pressure and strain sensing, tactile sensors are developed to sense multiple dimensional forces, including pressure, shear force, strain, and torsion. Tactile sensors with a single channel to respond to multi-dimensional forces were developed [97,98,99], as shown in Figure 4a. Although the readout circuit is simple, the force and torque vector cannot be obtained from the single output signal individually. Electronic skins with two strain sensing units perpendicular to each other to sense two-dimensional strains were developed [100,101,102,103], as shown in Figure 4b, and multi-dimensional force sensors were developed accordingly [104]. However, this type of design cannot distinguish planer forces with the same absolute value and opposite direction.

Various types of multi-dimensional tactile sensors able to distinguish forces with different directions and values were proposed [105,106,107]. A widely used method is to add a bump structure on the surface of four tactile sensing units to measure the torsion strains to determine planer shear forces [108], as shown in Figure 4c, and sensors based on resistive [109], capacitive [110], piezoelectric [111], transistor [112], microfluid [113], and conductive liquid [114] mechanisms have been demonstrated. Moreover, capacitive three-dimensional force sensors containing multiple capacitors in which the lower electrodes share the same upper electrode were developed [115], where the pressure and shear forces change the electrode distance and equivalent area of the four capacitors, respectively, and can be distinguished by the signal process, as shown in Figure 4d. Additionally, triboelectric multi-dimensional sensors with similar working mechanisms were proposed [116,117]. These kinds of multi-dimensional tactile sensor designs can be fabricated to be flexible, which can be highly conformal to human skin and can achieve comfortable wearable applications.

For most current multi-dimensional tactile sensor designs, the forces at X-, Y-, and Z- directions are not naturally decoupled; thus, the calibration is complicated for real cases when different forces in multiple directions are simultaneously applied. Therefore, a magnetic multi-dimensional tactile sensor was developed, where the normal and shear forces are naturally decoupled [72], as shown in Figure 4e. However, this design is sensitive to the magnetic field interference, and the hall sensor chip used in this design increases the device thickness. A three-dimensional resistive tactile sensor with five bumps responding to forces in five perpendicular directions [118] and a multi-directional flexible tactile sensor for pressure, shear forces, and strains decoupled sensing were proposed [119], where the output sensing signals in different directions are independent of one another, as shown in Figure 4f. Currently, multi-dimensional tactile sensors are required to be thinner and more flexible to be applied to electronic skin and HMI applications.

### 3.4. Wearing Fitness

**Figure 5:**
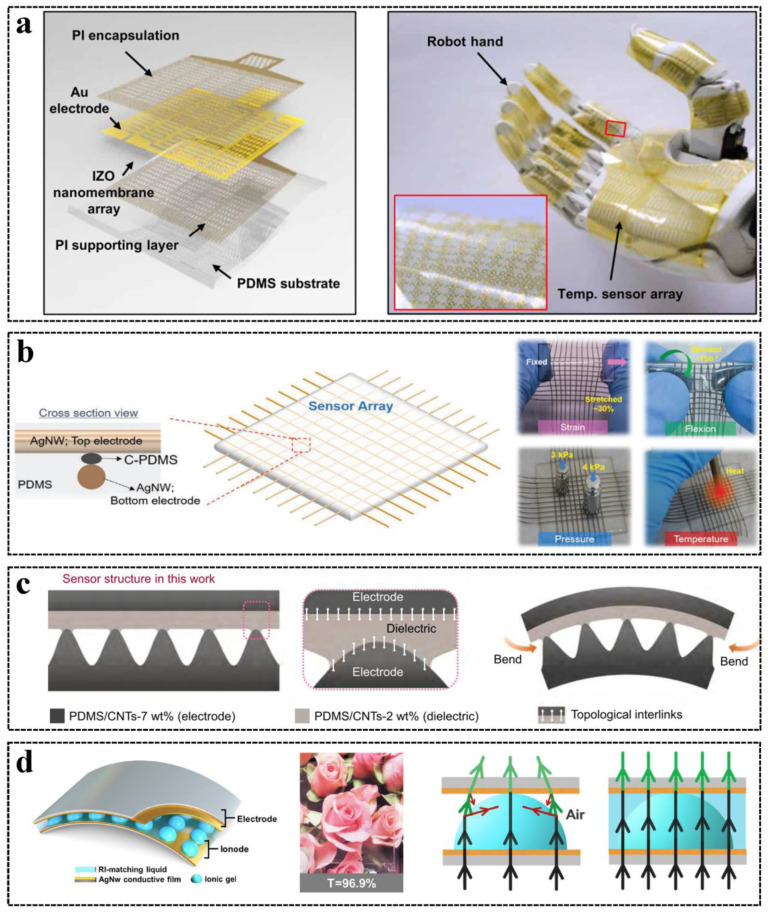
Tactile and force sensors with wearing fitness: (**a**) A stretchable tactile sensing array using patterned PI films. Reproduced with permission [120], Copyright 2019, American Association for the Advancement of Science. (**b**) A multimodal flexible sensing array using AgNW as the electrode. Reproduced with permission [11], Copyright 2020, Wiley-VCH. (**c**) A capacitive pressure sensor with stable pressure sensing property under bending conditions. Reproduced with permission [126], Copyright 2022, Springer Nature. (**d**) An iontronic capacitive pressure sensor filled with liquid to have high transparency. Reproduced with permission [130], Copyright 2022, Springer Nature.

With the development of wearable electronics, wearable human–machine interfaces with tactile and force sensors attached to clothing or human skin have been demonstrated [9]. Wearable human–machine interfaces should fit with the attached surface to recover the real tactile sense of the user and not disturb the user’s normal life. One of the specifications of wearing fitness is flexibility. Tactile and force sensors on human skin should be flexible and match the property of the skin to ensure comfortable wearing or even not be precepted by the users and adapt to the dynamic motion of the human body [120,121,122,123]. For HMIs that have single-channel tactile sensing units or multiple units distributed at many places, flexible nanomaterials are utilized for the tactile sensing of each unit [26,35,37,80,124]. For array-based HMIs, rigid electrodes and electrodes printed on Polyimide (PI) or Polyethylene Terephthalate (PET) films are non-stretchable and are hard to fit with human skin. Therefore, stretchable electrodes, created by printing metal electrodes on patterned plastic films, were developed and used for wearable tactile interfaces [120], as shown in Figure 5a. Apart from substrate patterning, another method for the stretchable array is to pattern flexible conductive materials as array electrodes, including graphene [43], liquid metal [125], and AgNWs [11] electrodes, as shown in Figure 5b. For wearable tactile sensors attached to high curvature surfaces, the bending can delaminate the sensor structure. Therefore, Zhang et al. developed a flexible capacitive pressure sensor with a quasi-homogeneous composition and interlinked interfaces [126], as shown in Figure 5c, where the pressure sensing is stable under bending conditions.

Transparency is also a crucial property for wearing fitness, and especially in intraocular applications, where the device should not block the line of sight [127]. Nanowires and few-layer graphene are commonly used electrodes and sensing materials due to their high transparency and conductance [128,129]. The active layer microstructures of pressure sensors are used to increase the sensitivity at the cost of losing transparency. A strategy of transparency recovery is to fill the microstructure with liquid that has a refractive index matching the active material [130], as shown in Figure 5d.

## 4. HMIs for Dexterous Robotic Manipulation

### 4.1. Multi-Channel Control

**Figure 6:**
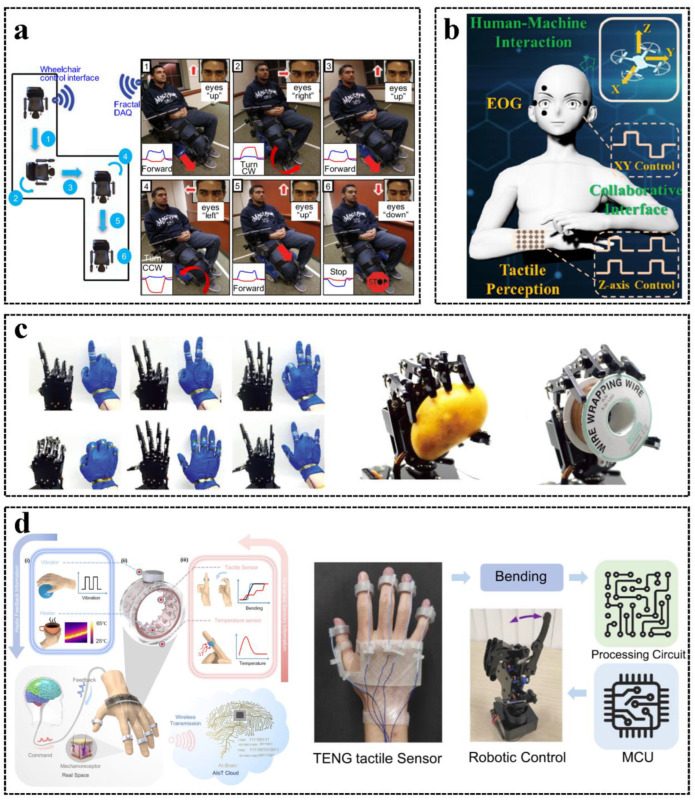
Multi-channel HMIs: (**a**) An HMI for wheelchair motion control via EOG signals. Reproduced with permission [131], Copyright 2017, Elsevier B.V. (**b**) An EOG and tactile collaborative HMI for 3D control. Reproduced with permission [43], Copyright 2022, American Chemical Society. (**c**) A wearable HMI for robot hand control. Reproduced with permission [80], Copyright 2022, Wiley-VCH. (**d**) Augmented rings as HMI for tactile perception and haptic feedback. (i) The feedback functionality. (ii) The ring structure. (iii) The sensing functionality. Reproduced with permission [20], Copyright 2022, Springer Nature.

Robot technology is a crucial field in the age of intelligence. Robots can reach hazardous places that humans need not attend and work personally. With various scenarios and HMI requirements, a diversity of brilliant solutions for multi-channel HMI for robot manipulation was proposed. A basic way to control a robot is to manipulate the robot’s motion in space. Mishra et al. proposed a convenient control HMI to manipulate the planer motion of a wheelchair by eye movement via multi-channel electrooculogram (EOG) signals [131], as shown in Figure 6a. To expand the HMI for robot control from two dimensions (2D) to three dimensions (3D), Xu et al. demonstrated an EOG and tactile collaborative HMIs to enable 3D control [43], as shown in Figure 6b. The EOG signals obtained by ultra-thin graphene electrodes are used for fast, convenient, and contactless 2D (XY-axis) interaction, and a flexible, ultra-thin, wearable haptic interface is utilized for 2D complex motion control and assisting the EOG signal to achieve *Z*-axis control in 3D control [43]. In addition, Chen et al. developed a self-powered triboelectric patch for a 3D robotic manipulator (the sensor patch, based on starch-based hydrogel, PDMS, and silicone rubber) that is composed of a 2D triboelectric sensor for in-plane robotic movement control and a 1D triboelectric sensor for out-of-plane robotic movement control [132]. However, these sensors are attached to one arm and palm, requiring two hands to achieve dexterous 3D control, which is not easy for people with disabled arms. The combination of EOG and the noisy brain activity signals such as electroencephalography (EEG) to generate robust control signals for HMI can completely free hands to achieve wireless interaction [133,134,135]. However, due to the low signal-to-noise ratio of EEG signals and the complexity of signal acquisition, further research and application are needed. Furthermore, the HMI based on the combination of electromyography (EMG), EEG, and EOG can be also used for hand-free wireless control of a machine such as a wheelchair [136,137,138].

The human hand possesses multiple arthroses, having a large degree of freedom (DOF). In order to make robots achieve a similar motion ability to the human hand, multi-channel sensing systems with units distributed at multiple places on the human skin or wearable devices have been proposed [139]. Tactile and force sensors are attached to hands or knuckles to monitor the finger bending and control the robot hand to respond to a different hand gesture or to grasp objects [37,66,74,80]. Tao et al. developed a triboelectric tactile sensor based on micro-pyramid-patterned double-network ionic organo-hydrogels [80]. The sensors were attached to knuckles, and the high sensitivity (45.97 mV/ Pa) and fast response (about 20 ms) enabled the sensors to capture the bending signal of the hand and realize robot hand control, as shown in Figure 6c. In order to enable HMI to sense multiple stimuli from the user, Kim et al. proposed a heterogenous tactile sensor that could sense stretching, bending, and compression individually, and it was demonstrated that the robot successfully responded to eight different types of tactile directives with higher than 95% accuracies [74]. The human–machine interactive system is an important component of intelligent robot technology, and there is a trend to combine tactile signals with multiple physicochemical signals for advanced HMI systems that establish loops of robot control and robot information feedback [17]. Sun et al. demonstrated a ring integrated with sensors (triboelectric sensors for tactile sensing and pyroelectric sensors for temperature perception) and haptic-feedback devices (vibrators for vibration feedback and nichrome heaters for thermal feedback) to build a closed-loop HMI that users can use to send directives to robots or virtual worlds and feedback information can be sent back to users [20], as shown in Figure 6d.

### 4.2. Machine Learning-Enhanced Control

An intelligent human–robot interactive system is a synthesis constructed by hardware sensors and software algorithms, and a suitable algorithm to analyze and recognize the signals obtained by the sensor can further enhance the power of the interactive system [140]. Many machine learning (ML) algorithms have been applied to process tactile information for object recognition [7], material sensing [141], touch modality classification [70], and HMI [17] applications for their powerful capability for pattern recognition, including convolutional neural networks (CNN) [7], support vector machines (SVM) [43], k-nearest neighbors (KNN) [17], etc. CNNs are good at extracting information from output signals and detecting multiple low-level features with high accuracy [140,142]. SVMs are one of the most efficient machine learning algorithms that are commonly used for classification, and SVMs can produce a unique solution, which makes SVMs more trustable over different samples compared to neural network algorithms [143]. KNNs have the advantages of their simplicity and superior accuracy in hand gesture recognition [17]. Many ML algorithms can be applied to tactile signal recognition, and the most suitable ML algorithm for a specific scenario should be given by testing and comparison.

**Figure 7:**
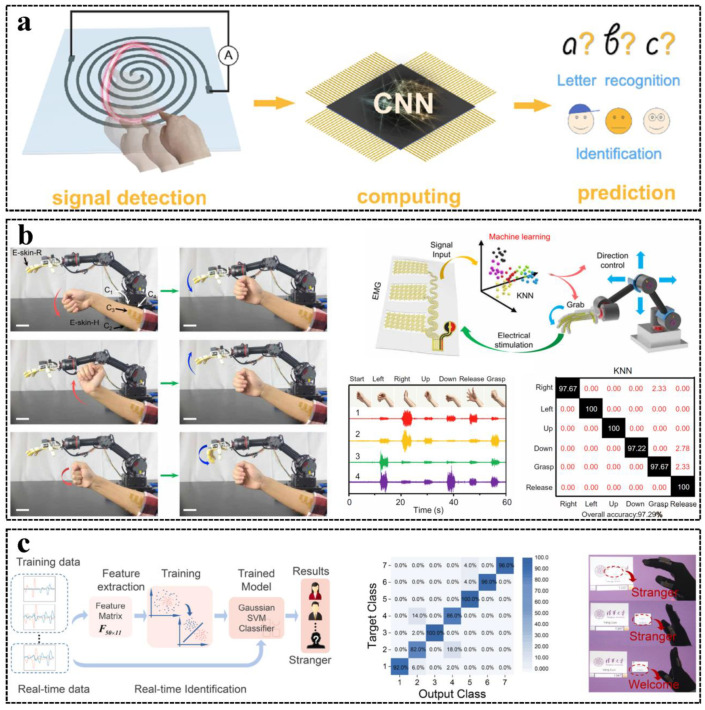
Machine-learning enhanced HMIs: (**a**) A handwriting input panel with 1D output using a CNN. Reproduced with permission [81], Copyright 2022, Elsevier Ltd. (**b**) A multimodal physicochemical sensing HMI using a KNN algorithm for gesture recognition. Reproduced with permission [17], Copyright 2022, American Association for the Advancement of Science. (**c**) A glove-based multi-dimensional HMI with user recognition functionality by an SVM algorithm. Reproduced with permission [146], Copyright 2021, Elsevier Ltd.

For HMI applications, ML algorithms are often introduced to accomplish the recognition task of many kinds of human motion patterns from tactile signals obtained by tactile and force sensors that are hard to directly understand by humans. For example, Xu et al. proposed an EOG and tactile perception collaborative interface and applied SVM to successfully recognize nine types of eye motion states with an accuracy of 92.6% [43]. Moreover, Hou et al. demonstrated a mouthguard integrated with an optoelectronic sensing system that can receive bite control signals [82]. An artificial neural network (ANN) algorithm was developed to extract the features of multi-channel bite signals and recognize the bite control directive from the user, and the accuracy was validated by wheelchair and virtual keyboard control tests (more than 94.2%). In order to reduce the number of readout circuits and simplify the electrode structure, Xu et al. demonstrated a handwriting panel with only one output channel [81], as shown in Figure 7a. A CNN algorithm was developed to achieve letter recognition from a single output channel, and the accuracy was 97%.

Gesture recognition can be achieved without ML algorithms by attaching tactile and force sensors on hands and fingers, but it may encumber the user’s normal life when using hands to work or grab; thus, HMI sensing systems attached on the arms or sleeves were developed to accurately recognize hand gestures by the output signals of arm muscle motions and ML algorithms [69,144]. For example, Yu et al. demonstrated a tactile sensor attached to the arm to recognize the gesture by a KNN algorithm for robot control with an accuracy of 97.29% [17], as shown in Figure 7b. For different hand gestures, the signals received by the tactile sensing units are not the same; thus, different gesture labels and the corresponding groups of signals received by the tactile sensing units obtained by experiments form a database to train the ML classifier algorithm. The resultant classifier is applied to real cases for gesture recognition, and the user can control the robot with different gestures. For a more intelligent and personalized system, the security of HMI is necessary. Therefore, HMIs with user identification functions have been demonstrated through tactile signal processing based on ML algorithms [145,146], as shown in Figure 7c.

## 5. HMIs for Virtual/Augmented Reality Applications

**Figure 8:**
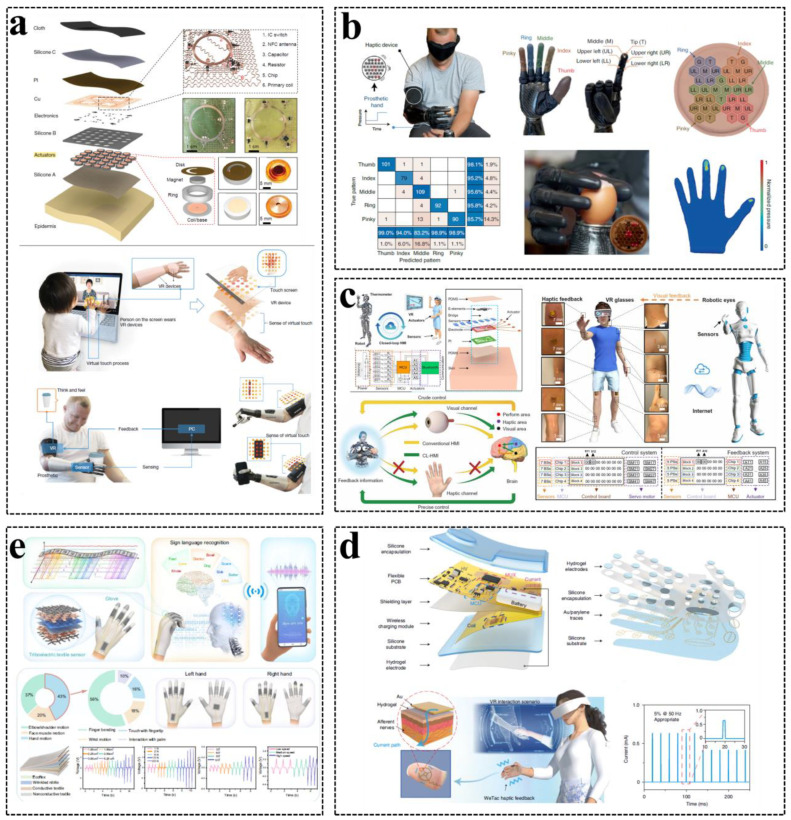
HMI for virtual/augmented reality applications: (**a**) Skin-integrated wireless HMI and feedback systems for AR/VR application. Reproduced with permission [147], Copyright 2019, Springer Nature. (**b**) Tactile interface as a sensory substitution for robot hand control feedback. Reproduced with permission [18], Copyright 2022, Springer Nature. (**c**) The closed-loop HMI in robotic VR applications. Reproduced with permission [9], Copyright 2022, American Association for the Advancement of Science. (**d**) A miniaturized wireless tactile system for personalized feedback with virtual objects. Reproduced with permission [148], Copyright 2022, Springer Nature. (**e**) A triboelectric smart glove for sign language recognition and VR communication. Reproduced with permission [149], Copyright 2021, Springer Nature.

Virtual reality (VR) and augmented reality (AR) technologies create human experiences related to the physical world by replicating visual and auditory stimuli of sensation. The most extensive VR and AR systems use head-mounted displays, accelerometers, and speakers as the basis for 3D computer-generated environments, which can exist independently or as an overlay of actual scenes. Therefore, eyes and ears are the key ways to obtain information in a virtual reality experience. The skin is the largest organ of the human body, but in VR technology, the skin is relatively undeveloped relative to the eyes and ears. Nowadays, tactile interaction is also gradually paid attention to. However, the current tactile devices used in VR and AR usually rely on the motor to exert skin vibration. Users need to wear heavy wires and batteries to achieve tactile interaction, which greatly limits the application. In order to immersively experience virtual reality scenes, more and more researchers developed skin-integrated interfaces as feedback and interactive applications of virtual reality technology, which has great application value in games, sports, medicine, and other fields [18,147]. For example, Rogers et al. developed skin-integrated wireless haptic interfaces for virtual and augmented reality. This system can receive instructions wirelessly and then overcome the cumbersome disadvantage through vibration simulation touch. As shown in Figure 8a, users can feel the virtual world, even the touch from relatives thousands of miles away, through a thin and soft device that can be attached to the skin [147]. Furthermore, they realized vibration tactile mode on a large area of skin in a single unit or through wireless coordination (Figure 8b). The Vibro-haptic actuators at a density of 0.73 actuators per square centimeter exceed the two-point discrimination threshold of mechanical sensation on the skin of almost all body regions except hands and faces [18]. Similarly, based on electronic skin, Xu et al. developed a closed-loop HMI for wireless motion capturing and haptic feedback via Bluetooth, Wireless Fidelity (Wi-Fi), and Internet (Figure 8c). The combination of visual and haptic VR by the closed-loop system can be integrated into the skin as a platform for the remote control of robots. They tested that a user wore four skin-integrated closed-loop HMI patches to control the 13-DOF humanoid robot and used corresponding pressure sensors to synchronously experience tactile information from the forearm, upper arm, thigh, thigh side, abdomen, and calf of the robot [9]. Yu et al. encoded hand tactile information through a skin-integrated wireless tactile interface to truly immerse virtual reality and augmented reality (Figure 8d). The wireless tactile system composed of a driver unit and the hydrogel-based electrodes hand patch can provide the user with personalized feedback on virtual object interactions by mapping the thresholds for different electrical parameters [148].

In addition to the electronic skin tactile system, some other tactile interfaces such as socks, gloves, rings, stylus, and so on are also used for AR/VR applications [20,68,149,150,151]. Lee et al. developed triboelectric smart socks for IoT-based gait analysis and established a digital human body system by mapping the physical signals collected by socks into the virtual space, which is beneficial for motion monitoring, medical care, recognition, and future smart home applications [68]. Wen et al. designed a triboelectric smart glove for sign language recognition and VR space bidirectional communication (Figure 8e). The language recognition and communication system composed of a smart glove, AI block, and back-end VR interface can independently recognize words and sentences with a high accuracy of 91.3% and 95% in a non-segmented framework, indicating the potential for advanced and practical language recognition. Furthermore, the VR platform can provide opportunities for speech/hearing-impaired people to directly use sign language to interact with non-sign language speakers [149]. Sun et al. designed an augmented tactile-perception and haptic-feedback ring for multi-modal immersive interaction, in which the triboelectric and pyroelectric sensors are used for tactile and temperature perception, and the vibrators and nichrome heaters are used for vibro- and thermo-haptic feedback. All the sensors are integrated in a minimalistic ring and driven by a custom IoT module, and the self-powered TENG and PVDF sensors can reduce the power consumption of the system for long-term use in wearable applications such as a manipulator [20]. Therefore, the multi-mode haptic perception interaction interface and feedback system can enable people to immerse themselves in AR/VR, and at the same time, these interaction systems can also solve the basic problems of the interaction and communication of some disabled people. In the future, wearable HMIs will break through the challenges, become more portable, convenient, and intelligent in design and more comprehensive and multi-functional in application scenarios, and will bring more comprehensive perception and feedback in the metaverse-based virtual society.

## 6. Conclusions and Perspectives

HMI is an important technology to establish communication between humans and robots, vehicles, and virtual worlds that has been widely applied to robot manipulation, handicapped equipment, social media, entertainment, etc. In recent years, HMI has become more intelligent, multi-functional, and miniaturized with advancements in material, structure, sensing system design, micro-nano processing technology, and recognition algorithm. This review discussed tactile and force sensing mechanisms for HMI applications and summarized the strategies used to enhance the performance of tactile and force sensors for advanced HMI. Robot control and AR/VR are two of the most common applications of HMI, and methods for advanced robot control and AR/VR were described in this review. In the future, advanced tactile and force sensors should be developed for HMIs that require high performance such as higher sensitivity, lower response time, larger linear detection range, higher wearing comfort, more intelligence, and multi-functionality. The tactile and force sensors for wearable HMIs should especially be properly designed to fit with the skin or clothing, such as having flexible, stretchable, and transparent properties. Furthermore, advanced, multi-functional, multi-sensory skin-integrated feedback systems should also be developed to achieve an immersive experience of the meta-universe, making the interaction between people and the virtual world more real, convenient, and free. Although many excellent skin-integrated sensors and other wearable devices for feedback systems have been reported in the literature, there is still no comprehensive wearable interaction and feedback system that can combine skin feedback with visual and auditory feedback. In the future, the multi-functional and multi-scene interactive feedback system can be used to replace the cumbersome interactive devices in today’s AR/VR systems, such as commercial controllers, interactive gloves, operating levers, etc. Additionally, the proper distribution and system design of tactile sensing units for effective and accurate tactile signal collection for specific applications ought to be further developed, and signal processing software systems compatible with the tactile sensing hardware should be achieved and applied for HMIs.

## Figures and Tables

**Table 1:** Summary of typical tactile and force sensors for human–machine interaction.

Ref.	Sensor Types	Sensor Features	Sensitivity	Detection Range	Applications
[28]	Resistive pressure sensor	Ag nanowires, carbon fabric, hetero-contact microstructure	4.1 kPa^−1^ in 0–10 kPa, 1.2 kPa^−1^ in 10–65 kPa	65 kPa	Virtual reality
[26]	Resistive pressure sensor	SWCNTs, pyramid structure	−11,570.9 Ω/N in 0–1.8 N −62.5 Ω/N in 3–10 N	10 N	Interactive games
[9]	Resistive bending and pressure sensor	Fully flexible configurations with skin-integrated elements	~0.037 degree^−1^ (bending) ~0.0058 kPa^−1^ (pressure)	~150° (bending) ~120.5 kPa (pressure)	Robotic virtual reality
[37]	Resistive strain sensor	Graphene/Ecoflex, multiscale/hierarchical wrinkles	GF = 1078.1	650% stretchability	Robot hand control
[76]	Resistive strain sensor	Biocompatible solderable graphene, all printed PI/Graphene/Ag/PI/Ag/PI	/	/	Robot hand control
[43]	Resistive strain sensor, capacitive sensor	Honeycomb graphene electrodes, laser-induced graphene array	Resistive strain sensor: GF = 41 in 0–50% strain, Capacitive sensor: 1.428 kPa^−1^ in 0–300 Pa and 0.085 kPa^−1^ in 300–3300 Pa	1000% strain sensor stretchability, 3300 Pa pressure sensor range	Three-dimensional HMI
[50]	Capacitive sensor	PVDF dielectric, convex microarrays	30.2 kPa^−1^ in 0–130 Pa and 0.47 kPa^−1^ in 0.13–10 kPa	10 kPa	Physiological signal and grabbing monitoring for HMI
[52]	Capacitive pressure and proximity sensor	High aspect ratio cellulose fibers, CNTs.	Proximity sensitivity: <5 fF/mm, Contact sensitivity: ~110 pF/N in 0–0.04 N, and ~1 pF/N in 0.6–1.5 N	Proximity detection: 300 mm, contact detection: 2 N	Smart pad and human gesture recognition for HMI
[77]	Capacitive sensor	Ionic hydrogels and Ag nanofibers	GF = 165	1000% stretchability	On-skin monitoring for HMI
[60]	Piezoelectric sensor	All-inorganic Sm: PMN-PT	5.86 V/N $V_{\mathrm{oc}}=6\;V\;\mathrm{and}\;J_{\mathrm{sc}}=150\;\mu A/{\mathrm{cm}}^{2}\;\mathrm{under}\;60{}^{\circ}$ bending	~1.45 N	A controller using body motion and a touchscreen
[78]	Piezoelectric sensor	MDABCO-NH_4_I_3_	V_oc_ = 15.9 V and I_sc_ = 54.5 nA under 0.55% strain	~0.55% strain	Gesture-controlled HMI
[79]	Piezoelectric sensor	WS_2_ nanosheets	V_oc_ = 65 mV and I_sc_ = 325 pA under 1.56% strain	~3.5% strain	Gesture-controlled HMI
[66]	Triboelectric sensor	Ni-fabric and PTFE films as the L-TENG, and Ni-fabric on PET substrate as the T-TENG	/	/	Robot hand control
[80]	Triboelectric sensor	Micro-pyramid-patterned double-network ionic organo-hydrogels	45.97 mV/Pa	~1 kPa	Robot hand control
[81]	Triboelectric sensor	Spiral carbon grease (CG) electrodes sandwiched by PU	/	/	Handwriting input panel with 1D output
[68]	Triboelectric sensor	Nitrile thin film, patterned frustum structure silicon rubber	~1.2 V/kPa within 42 kPa	>200 kPa	Smart socks for virtual reality
[82]	Optical sensor	Mechanoluminescent phosphors of ZnS: M (M = Mn2+ or Cu2+)@Al2O3 particles	20 intensity counts/N	60 N	Bite-controlled robot navigation
[74]	Tactile sensor combining optical, microfluidic, and resistive sensing	Elastomer waveguide with LED and PD, RTIL, Medtex P130 fabric layer	Stretching: 0.0208%^−1^, bending: 50.26 mm^−1^, compression: 0.021 kPa^−1^	Stretching: 50%, Bending: 0.05 mm^−1^, compression: 292 kPa	Robot hand control

## Data Availability

Not applicable.
